# Development of a New Electrochemical Sensor Based on Molecularly Imprinted Biopolymer for Determination of 4,4′-Methylene Diphenyl Diamine

**DOI:** 10.3390/s23010046

**Published:** 2022-12-21

**Authors:** Masoud Ghaani, Duygu Büyüktaş, Daniele Carullo, Stefano Farris

**Affiliations:** 1 DeFENS, Department of Food, Environmental, and Nutritional Sciences, Food Packaging Lab., University of Milan, via Celoria 2—I, 20133 Milan, Italy; 2 Department of Food Engineering, Faculty of Engineering, Izmir Institute of Technology, Gülbahçe Köyü, Urla, Izmir 35430, Turkey; 3 INSTM, National Consortium of Materials Science and Technology, Local Unit University of Milan, Via Celoria 2—I, 20133 Milan, Italy

**Keywords:** 4,4′-methylene diphenyl diamine, primary aromatic amine, electrochemical sensor, chitosan, molecularly imprinted polymer

## Abstract

A new molecularly imprinted electrochemical sensor was proposed to determine 4,4′-methylene diphenyl diamine (MDA) using molecularly imprinted polymer–multiwalled carbon nanotubes modified glassy carbon electrode (MIP/MWCNTs/GCE). GCE was coated by MWCNTs (MWCNTs/GCE) because of their antifouling qualities and in order to improve the sensor sensitivity. To make the whole sensor, a polymeric film made up of chitosan nanoparticles was electrodeposited by the cyclic voltammetry method on the surface of MWCNTs/GCE in the presence of MDA as a template. Different parameters such as scan cycles, elution time, incubation time, molar ratio of template molecules to functional monomers, and pH were optimized to increase the performance of the MIP sensor. With a detection limit of 15 nM, a linear response to MDA was seen in the concentration range of 0.5–100 µM. The imprinting factor (IF) of the proposed sensor was also calculated at around 3.66, demonstrating the extremely high recognition performance of a MIP/MWCNT-modified electrode. Moreover, the sensor exhibited good reproducibility and selectivity. Finally, the proposed sensor was efficiently used to determine MDA in real samples with satisfactory recoveries ranging from 94.10% to 106.76%.

## 1. Introduction

In recent years, because of the growing need to combine different functional requirements in food packaging materials, the usage of multilayer materials has attracted much attention [1]. Polyurethane (PU) adhesives are one of the most popular groups of adhesives used in film–film food packaging materials [2]. These adhesives are the products of the reaction of polyols (usually polyester polyol or polyethylene glycol) with diisocyanates: Ar(NCO)_2_+R(OH)_2_→ 6-(OCOHNArNHCOOR)- [3]. Primary aromatic amines (PAAs), which are “possibly carcinogenic to humans,” are substances that can migrate from food packaging materials consisting of PU adhesives into foodstuffs [4]. The presence of PAAs in the food packages can be explained by two main reasons: (a) some residual isocyanic monomers that may remain in PU adhesives after the curing process can react with the moisture that is present in food packages and yield PAAs; (b) after some thermal processes (e.g., pasteurization and sterilization), urethane linkage may break, yielding neoformed isocyanic monomers that can come into contact with the moisture inside the food package, thus forming PAAs [2]. According to European Regulation 10/2011, plastic material and articles shall not release PAAs in detectable quantity in food or food simulants. The limit is set to 0.01 mg of substances per kg of food or food simulant, and it relates to the sum of primary aromatic amines (ANL, 2-4TDA, 2-6TDA, 4-4MDA, 1-5DAN, m-PDA, 3-3DCB, and 4-4DPE) released [5]. In the past few years, the accurate quantification of PAAs has become a critical topic in the food packaging area. Packaging companies are willing to use methods that are cheap and, at the same time, sensitive and selective enough. Lately, electrochemical sensors have been known as selective, sensitive, and reliable devices with some extra advantages over other analytical techniques, advantages such as ease of use, lower cost, and faster response time [6,7,8,9,10].

Sensors are known as small devices resulting from incorporating a recognition element with a signal transducer that can be used for the direct measurement of the analyte in the sample matrix. One of the most important subclasses of sensors is electrochemical sensors, in which an electrode is used as the transduction element [11]. Bare electrodes are not appropriate options for fabricating electrochemical sensors, because of their low surface area and electrode-fouling phenomenon, which may happen during the electroanalytical analysis, and that can negatively affect the analytical characteristics of a sensor (e.g., reproducibility, overall reliability, and sensitivity). In recent years, modified electrodes have been suggested to overcome the deficiencies of bare electrodes. Carbon-based materials with large surface areas (e.g., graphene and carbon nanotubes) are well suited to modify bare electrodes because of electrocatalytic properties and fouling resistance [12]. Another group of materials that has received considerable attention for increasing the selectivity and sensitivity of the sensor is represented by the molecularly imprinted polymers (MIPs) [13].

Molecular imprinting is a well-established and simple method to fabricate materials with recognition sites with complementarities in size, shape, and functional groups with the template molecule. MIP materials can mimic natural receptor systems (antibodies, enzymes, and hormones) to bind with a specific target analyte [14,15]. The main steps for MIPs synthesis are as follows: (1) a combination of template molecules with the functional monomers to make a composite via noncovalent bonds or covalent in solution; (2) polymerizing the complex with initiators and cross-linkers under photo-/thermal conditions; (3) a template removing the use of solvent elution because the analyte has higher solubility in the solvent [16]. MIPs can bind specifically to the original and related template molecules while also possessing tolerance to mechanical stress, temperature, pH, etc. [17]. MIP materials have attracted much attention in recent years because of their great potential for electrochemical applications. In particular, MIPs can advantageously be used as functionalized polymers for making more-sensitive and more-selective electrochemical sensors [16,18,19].

By combining electrochemical sensors and molecular imprinting, molecularly imprinted electrochemical (MIEC) sensors can be prepared. This type of sensor has some crucial properties, such as high sensitivity, selectivity, chemical stability, and ease of preparation [20]. Among numerous methods, electropolymerization is one of the best techniques for fabricating rigid, uniform, and compact MIP films on the electrode surface in a relatively simple fashion [21]. Moreover, MIP films prepared by electropolymerization have high stability, electrocatalytic activity, and conductivity, which can improve the sensitivity and selectivity of the sensor [13]. The easiest way to increase the number of effective imprinted sites on the sensor surface is to assemble the nanoparticles at the surface of the electrode to increase its surface area [22].

Chitosan (CS) is a linear polysaccharide made up of β(1→4)-linked D-glucosamine residues with a different number of randomly located N-acetyl-glucosamine groups [23]. CS can be prepared by the deacetylation of chitin, isolated from the cell walls of many fungi and shells of crustaceans [24]. This biopolymer has some unique properties, such as biodegradability, biocompatibility, film-forming ability, nontoxicity, high mechanical strength, and high adsorption and adhesion properties [25,26]. There are diverse approaches to producing CS films, such as drop casting, nanoimprinting, spin coating, and electrodeposition. The latter, in particular, offers better overall deposition control over the other approaches, which can also benefit from the pH-dependent solubility of this biopolymer [27].

The main goal of this investigation is to prepare a new, selective, and sensitive analytical device for determining 4,4′-methylene diphenyl diamine (MDA), one of the most important PAAs that can migrate from PU-based multilayer packaging materials to the packaged food. Conductive MWCNTs were used to increase the electrode’s surface area and improve its resistance to fouling. We also used the electropolymerization technique to fabricate a chitosan molecularly imprinted polymer (MIP) layer on the electrode’s surface to increase the sensor’s selectivity. The analytical performance of the sensor was thoroughly investigated by differential pulse voltammetry (DPV). The experimental parameters possibly affecting the performance of the MIP sensor were studied and optimized, while the potential application of the developed sensor in real samples was evaluated by preliminary trials.

## 2. Materials and Methods

### 2.1. Chemicals and Apparatus

First, 4,4′-methylene diphenyl diamine (analytical grade 98%, molar mass 198.26 g mol-1), multiwalled carbon nanotube (≥98% carbon basis), chitosan (≥75%, deacetylated), acetic acid (99%), boric acid (99.99% trace metals basis), phosphoric acid (85%–90%), phosphate buffer solution (pH 7.0), and N,N-dimethylformamide (99.8%) (DMF) were purchased from Sigma Aldrich (Milan, Italy). Irganox 1010 (Pentaerythritol Tetrakis(3-(3,5-di-tert-butyl-4-hydroxyphenyl)propionate)) and Irgafos 168 (Tris (2,4-ditert-butylphenyl)phosphite) were bought from BASF (Pontecchio Marconi, Italy). Alumina powder, 0.05 µm, was purchased from EMS (Hatfield, PA, USA). The Britton–Robinson (B-R) universal buffer (0.04 M acetic acid, 0.04 M boric acid, and 0.04 M phosphoric acid) was made using deionized water.

All the electrochemical experiments were carried out by an Autolab PGSTAT 302N potentiostat (Metrohm, Herisau, Switzerland). The applied three-electrode electrochemical cell was equipped with a MIP/MWCNTs-CS/GC electrode as a working electrode, a platinum electrode as an auxiliary electrode, and an Ag/AgCl electrode as a reference electrode. The pH was measured with a pH meter BASIC 20 + (Crison Instruments, S.A. Barcelona, Spain). All the measurements were performed at room temperature (25 ± 2.5 °C). The drying of the modifications was performed by using an infrared lamp (type B, 1440 W, Helios Italquartz srl, Cambiago, Italy).

### 2.2. Fabrication of MWCNTs Modified GCE

MWCNTs (0.5 mg) was added into 1 g DMF solution, and the mixture was ultrasonicated for 3 min to form a homogeneous MWCNTs suspension.

A GCE was polished with an Al_2_O_3_ slurry and then rinsed with doubly distilled water. To fabricate the MWCNTs modified GCE (MWCNT/GCE), 15.0 µL of MWCNTs-DMF solution were placed directly onto the GCE surface and dried with an infrared lamp for 10 min to form a MWCNTs layer.

### 2.3. Preparation of MIP and Nonimprinted Modified Electrodes

**Scheme 1:**
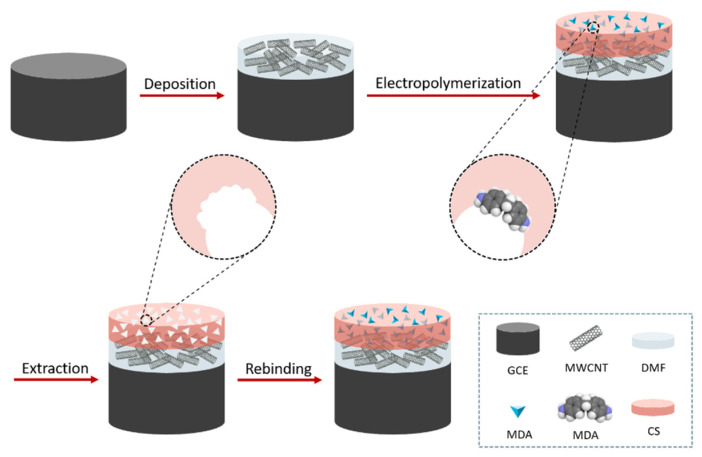
Schematic illustration of the preparation of the MIP/MWCNTs/GCE.

Chitosan powder (0.1 g) was added to hydrochloric acid 1 M (0.52 g) and water (9.38 g). The mixture was then left under stirring for 15 min at room temperature. A 10 mM solution of MDA was separately prepared in ethanol. Subsequently, the chitosan solution, MDA, the supporting electrolyte, and water were mixed to prepare the modifying solution. MIPs were obtained by electrodepositing chitosan and the analyte (MDA) on the surface of the MWCNTs/GCE. The MIP-modified electrode, denoted as MIP/MWCNTs/GCE, was prepared by cyclic voltammetry (CV) in the range of -0.5 V to 1.5 V and at a scan rate of 50 mV S^–1^ in the modifying solution [26]. After the electrodeposition, the MIP/MWCNTs/GCE was immersed in an eluent solution (0.5 mM NaOH solution containing 200 µL of ethanol) to remove the MDA-templating molecules. A schematic sketch of the preparation of MIP/MWCNTs/GCE is illustrated in Scheme 1. A nonimprinted polymer (NIP) sensor, denoted as NIP/MWCNTs/GCE, was prepared similarly to the MIP/MWCNTs/GCE, except that the template molecules were absent in the electrodeposition step.

### 2.4. Morphological Characterization of the Electrode Surface

A field emission–scanning electron microscope (FE-SEM), Hitachi S-4800 (Schaumburg, IL), was used for electrode-surface imaging. MWCNTs-coated GCE specimens were mounted with carbon tape on stubs, and their surfaces were observed after sputtering with Pt/Pd (60/40) under argon for 20 s at a current of 80 mA. The samples were observed using an acceleration voltage of 1-5 kV and an electrode current of 10 µA.

### 2.5. Real Sample Analysis

Thermo-sealed bags of 1 dm^2^ of surface area per side were prepared by using a multilayer packaging material (Castagna Univel Spa, Guardamiglio, Italy) consisting of polyethylene terephthalate (PET, 12 μm thick), olyvinylidene chloride coating (PVDC, 6 μm thick), and low-density polyethylene (LDPE, 50 μm thick), whereby a PU adhesive was used to join PET to the remaining part of the film. To investigate the migration of PAAs from PU-based multilayer packaging materials in the worst-case condition, each bag was filled with 100 mL of food simulant B (i.e., acetic acid water solution, 3 *w*/*v*%) [28]. The test was conducted at 121 °C for 20 min in an autoclave (Asal 760, Steroglass srl, Perugia, Italy). After this time, 10 mL of simulant B was diluted with 10 mL B-R buffer solution, followed by the addition of specific amounts of MDA monitored by DPV. From the quantitative determination of MDA, the final recovery (%) was determined.

## 3. Result and Discussion

### 3.1. Morphological Characterization of Modified GCEs

**Figure 1:**
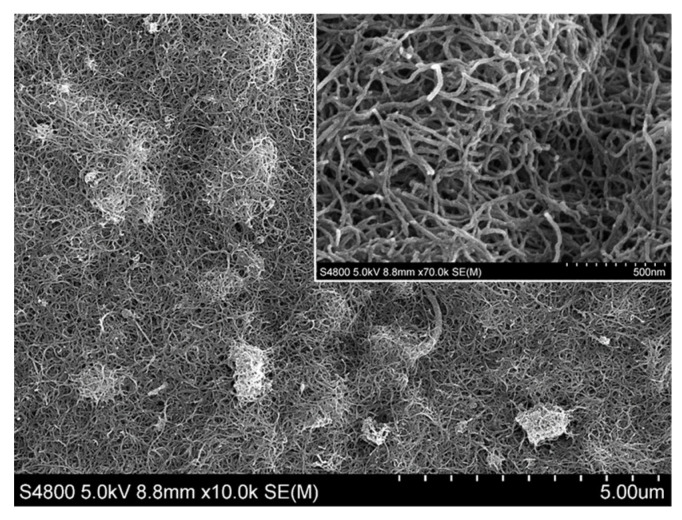
SEM image of multiwalled carbon nanotube–modified glassy carbon electrode (MWCNTs/GCE). A zoomed-in area is shown in the inset.

Scanning electron microscopy (SEM) was used to investigate the surface morphology of the electrode modified with MWCNTs (MWCNTs/GCE). Figure 1 shows the MWCNTs on the surface of the electrode at two magnifications. As can be seen, the nanotubes were evenly distributed throughout the surface of the electrode, thereby contributing to a noticeable increase in the surface area of the electrode exposed to the surrounding medium.

### 3.2. Electrochemical Responses

**Figure 2:**
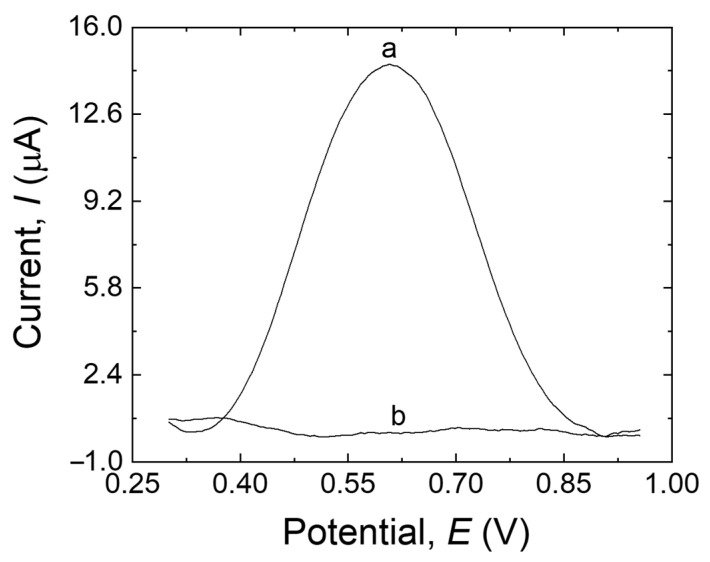
DPV responses of the MIP/MWCNTs/GCE before extraction (trace a) and after extraction (trace b) in 20 mL B-R buffer (pH 10.0).

Figure 2 shows the DPV responses of the MIP/MWCNTs/GCE with and without the template. An obvious current peak was detected in the presence of the template encased in the main biopolymer network (trace a). However, no peak was observed when the MIP/MWCNTs/GCE was eluted with a solution of ethanol and NaOH (trace b). This demonstrated that the ethanol-NaOH solution is an efficient solvent for extracting MDA, thus generating the final MIP structure.

**Figure 3:**
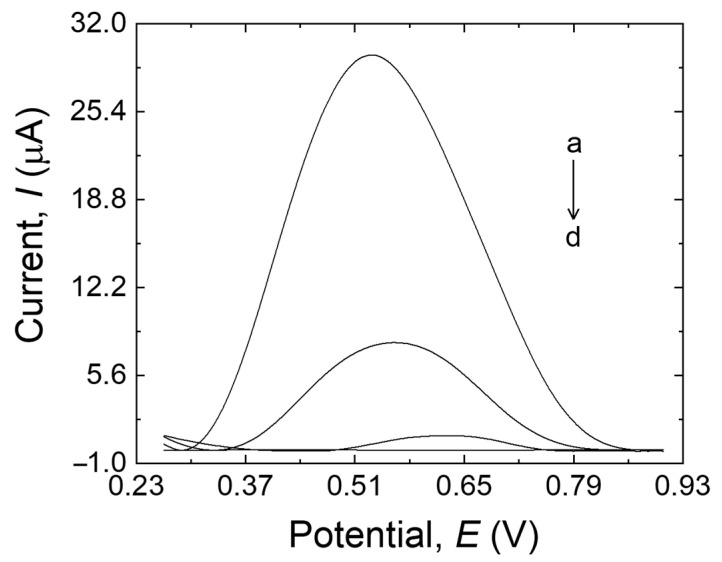
MIP/MWCNTs/GCE (a), NIP/MWCNTs/GCE (b), bare GCE (c), and MIP/GCE (d) in 20 mL B-R buffer (pH 10.0) containing 500 µM MDA.

The electrochemical behavior of MDA at the surface of MIP/MWCNTs/GCE (a), NIP/MWCNTs/GCE (b), MIP/GCE (c), and bare electrode (d) was investigated by DPV, as shown in Figure 3. The peak current at the modified/unmodified GCE varied according to the following order: MIP/GCE < bare electrode < NIP/MWCNTs/GCE < MIP/MWCNTs/GCE. The bare electrode showed a peak with a higher current than MIP/GCE (trace c–d, Figure 3), which can be explained by the fouling that occurred during the electropolymerization on the surface of the bare electrode as well as by the low sensitivity of the bare electrode, possibly due to the low surface area of the electrode. NIP/MWCNTs/GCE (trace b, Figure 3) showed a higher peak current than the electrodes discussed before. This could be attributed to the antifouling effect of MWCNTs and also to the high conductivity and surface area of the nanotubes, which can result in increased sensitivity to the sensor. Furthermore, the peak current at the MIP/MWCNTs/GCE (trace a, Figure 3) is higher than the others, which suggests that MDA molecules have been extracted from the MIP structure and that the cavities were correctly made in the MIP structure. The MIP’s nanoporous structure made it easier for MDA molecules to move to the electrode’s surface, accelerating the oxidation of the analyte and boosting its detection. These results demonstrate that MWCNTs can amplify the response signal while using the imprinting process to identify MDA. The electrocatalytic oxidation characteristics of MDA at various electrode surfaces at pH 10.0 are summarized in Table 1.

### 3.3. Optimization of Analytical Conditions

#### 3.3.1. Effect of Scan Cycles

**Figure 4:**
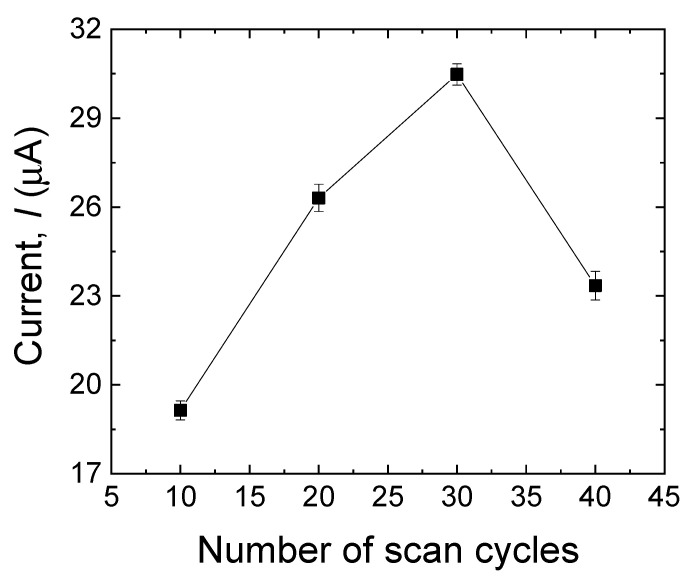
Effect of a different number of scan cycles in the electropolymerization process on the anodic peak currents of MDA (500 µM). Error bars are calculated from three independent replicates.

One of the most important factors that should be considered while dealing with MIPs is the film thickness [20]. More specifically, in the case of very thin imprinted membranes, the main drawback is associated with the low number of imprinted sites that can be formed on the surface of the electrode, which may result in low sensitivity in the sensor. On the other hand, when the imprinted layer is excessively thick, the sensor may have limited sensitivity, which could be because of two main reasons: (i) it is almost impossible to remove the template molecules that are located in the bulk of the membrane; (ii) the high mass-transfer resistance, accessing the imprinted sites located in the bulk of the membrane is hard for the target analyte. The simplest way to control the polymer membrane’s thickness is to monitor the number of scan cycles throughout the electropolymerization process [29]. In this work, various scanning cycles (10, 20, 30, and 40) were used for the electrodeposition process to control the thickness of the imprinted film. As shown in Figure 4, the peak current response of the MIP sensor toward MDA increased with the cycle numbers, reaching its maximum: 30 cycles. The steeping part of the plot is plausibly due to the progressive increase in the MDA-binding sites, whereas the descending part of the plot might be due to the thick sensing layer with fewer available imprinted sites. Because the peak current response reached its maximum at 30 scanning cycles, this setup was selected to obtain the best performance of the MIP layer [30,31].

#### 3.3.2. Effect of Elution Time

**Figure 5:**
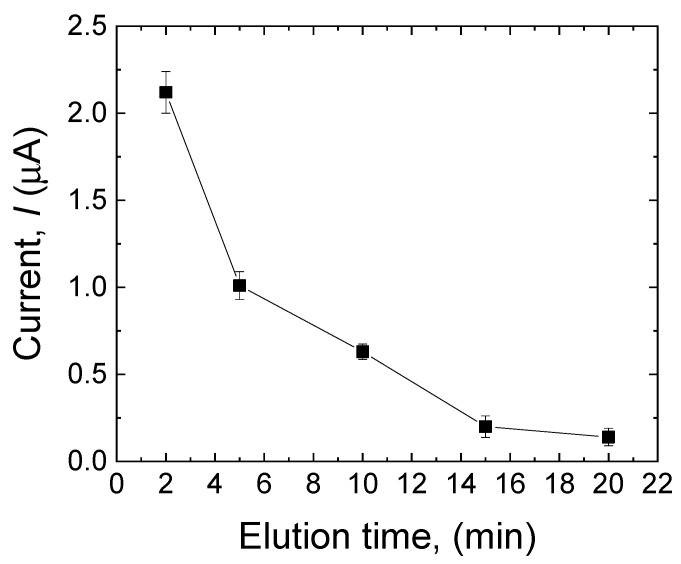
Effect of elution time (2 min, 5 min, 10 min, 15 min, and 20 min) on the current response of the sensor to MDA in the MIP layer. Error bars are calculated from three independent replicates.

The elution of the template is a crucial step during the MIP formation, as it can directly affect the sensor’s selectivity and sensitivity by successfully removing the template from the surface of the electrode and making the cavities instead. The template removal was performed in 0.5 mM NaOH solution containing 200 µL of ethanol for 2 min, 5 min, 10 min, 15 min, and 20 min. As shown in Figure 5, after 20 min, the peak current approached zero, which means that almost all the MDA molecules were removed from the surface of the electrode, and the electrode has the highest porosity to be used in the solution containing the target analyte. Therefore, an elution time of 20 min was selected for this work.

#### 3.3.3. Effect of Incubation Time

**Figure 6:**
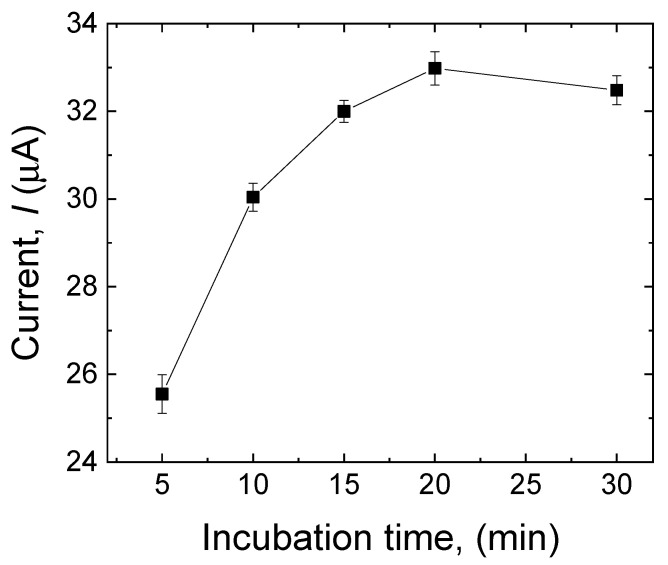
Effect of incubation time (5 min, 10 min, 15 min, 20 min, and 30 min) on the current response of the sensor to MDA (500 µM). Error bars are calculated from three independent replicates.

The effect of the incubation time on the sensor’s response toward MDA was investigated to optimize the adsorption of the analyte in the MIP layer. The modified electrode (after the template extraction) was incubated in 500 µM MDA for 5 min, 10 min, 15 min, 20 min, and 30 min. As shown in Figure 6, the peak current progressively increased with prolonged incubation time. It achieved its highest value after 20 min, indicating that in this period, the highest amount of MDA adsorption had been reached. After 20 min, the current response slightly decreased, suggesting that the MIP layer approached its saturation. Consequently, the incubation time of the MIP sensor was set at 20 min.

#### 3.3.4. Effect of the Template–Biopolymer Molar Ratio

**Figure 7:**
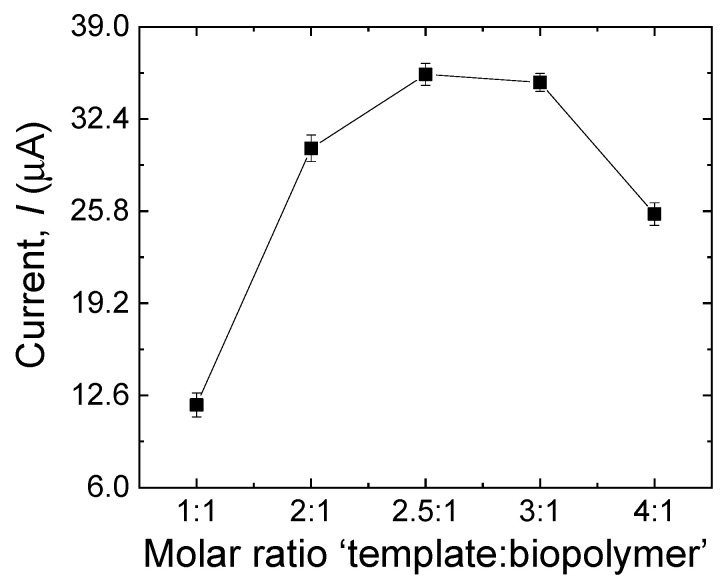
Effect of the template–biopolymer molar ratio during the electropolymerization process on the current response of the sensor to MDA (500 µM). Error bars are calculated from three independent replicates.

The performance of the MIP sensor is also affected by the molar ratio between the template and the biopolymer in the polymerization process [29]. The molar ratio of MDA to CS was investigated by varying the MDA–CS molar ratio as follows: 1:1, 2:1, 2.5:1, 3:1, and 4:1. As shown in Figure 7, the peak current increased as the ratio increased, and a maximum value was reached at 2.5:1, after which the peak current decreased gradually. According to this result, it can be said that the sensor is less sensitive at lower template concentrations, in agreement with a smaller number of recognition sites made in the biopolymer matrix. Concurrently, low sensitivity was observed for high concentrations of the template, which can be explained by the tendency of MDA molecules to make a complex, thus reducing the probability for the template molecule to be entrapped in the tri-dimensional biopolymer matrix [32].

#### 3.3.5. Effect of the pH

**Figure 8:**
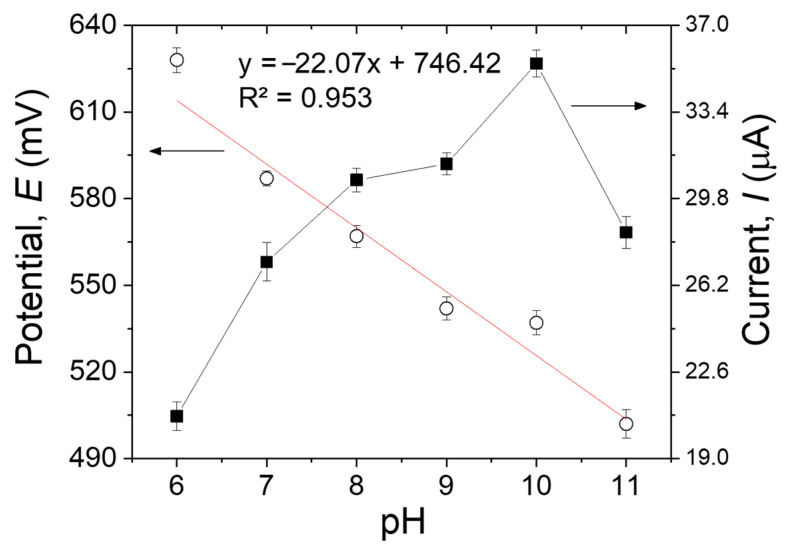
The pH effects on the anodic peak potentials and currents of 500 µM of MDA in B-R buffer solution on the MIP/MWCNTs/GCE. Error bars are calculated from three independent replicates.

One of the most important parameters on the performance of the MIP sensors is the pH, not only because it influences the oxidation rate but also because it may affect both the shape of the target molecules and the function and structure of the imprinted polymer [22]. The impact of the pH on the peak potential (*E*_pa_) and peak current (*I*_pa_) was investigated by DPV using 500 µM of MDA in the pH range of 6-11 (Figure 8). As the pH increased from 6 to 10, the peak current of the imprinted sensor increased progressively. Conversely, when the pH changed from 10 to 11, the peak current declined, probably because of the impact of the high pH on the oxidation of MDA on the surface of the modified electrode [33]. Hence, the B-R buffer solution with pH 10.0 was selected as the best electrolyte to achieve the best sensitivity in all the measurements. The linear relationship between *E*_pa_ and pH can be expressed by the following equation: *E*_pa_ (V) = −22.07 pH + 746.42. The potential negatively shifts by 22.07 mV per pH unit, demonstrating that one proton every two electrons was involved in the electrochemical reaction [34].

### 3.4. Performance of the Imprinted MIP/MWCNTs/GCE Sensor

#### 3.4.1. Sensitivity

**Figure 9:**
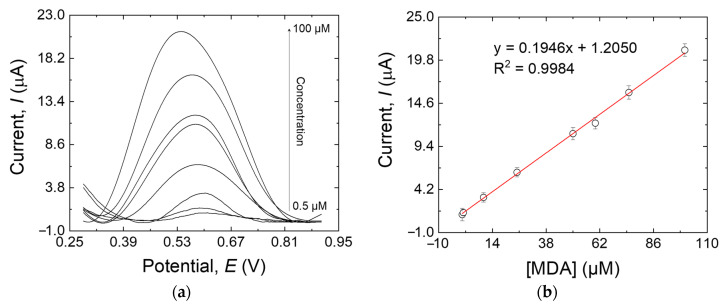
(**a**) DPV responses of the MIP/MWCNTs/GCE at different MDA concentrations: 0.5, 1, 10, 10, 25, 50, 60, 75, and 100 µM; (**b**) the calibration line of the response peak current versus the MDA concentration. Error bars are calculated from three independent replicates.

The sensitivity of the MIP/MWCNT-modified electrode to MDA was assessed by DPV according to the optimized conditions discussed earlier. Figure 9a shows that the oxidation peak was centered at +0.53 V, and a proportional relationship exists between the MDA concentration and the peak current intensity. A linear calibration curve was obtained in the range of 0.5 µM to 100 µM (Figure 9b) with the following linear regression equation: *I*_p_ = 0.1946x + 1.2050 (R^2^ = 0.9984). The ultimate limit for the detection of the sensor was calculated to be 15 nM, according to the method reported by Skoog et al. [35],

LOD = (3 × S_bl_)/m (1)

In this equation, S_bl_ is the standard deviation of the blank response (0.001 μA in this work), whereas m is the slope of the calibration plot (0.1946 μA μM^−1^).

Another important parameter that is used to describe the analytical performance of the electrode is the imprinting factor (IF), which shows the recognition performance of the imprinted sensor. This factor can be calculated by the fitting parameters of *I*_pm_.

IF = *I*_pm_(MIP)/*I*_pm_(NIP) (2)

*I*_pm_ (MIP) is the peak current of the MIP/MWCNTs/GCE toward the analyte, and *I*_pm_ (NIP) is related to the NIP/MWCNTs/GCE. In this experiment, IF is equal to 3.66, which shows a high selectivity performance of the MIP-modified electrode. This is another piece of indirect evidence of the presence of cavities on the surface of the electrode [36]. Our examination of the literature shows that just one paper reported employing a graphene-based MIP electrochemical sensor to measure MDA concentration. The linear range and slope of the calibration curve of that sensor were 1–15 µM and 0.0352, respectively [34]. These two items clearly show the advantages of our sensor over that in the previous work.

#### 3.4.2. Selectivity and Reproducibility

Interference in the measurement of the target analyte due to other electroactive species is a problem that may arise during the analyte detection in real samples. The selectivity of the MIP sensor toward MDA was investigated by assessing the interference of some potential compounds (Aniline, TDA, IRGAFOS 168, IRGANOX 1010) that may be present in real food packaging samples as a consequence of the unwanted migration of additives/compounds present in the packaging material. The results (data not shown) demonstrated that these compounds did not affect the MDA measurement.

To check the sensor’s reproducibility, three MIP/MWCNTs/GCE sensors were prepared independently and used in the same way to determine 50 µM MDA. The relative standard deviation (RSD) for three successive runs was about 4.13%. This result indicates satisfactory reproducibility for the proposed electrode.

#### 3.4.3. Real Sample Analysis

The practical application of the MIP/MWCNTs/GCE was evaluated by using the modified electrode to determine MDA in real samples. Given that no MDA was detected in the sample, the standard addition method was used to evaluate the potential of MIP/MWCNTs/GCE in real applications. The results obtained by the proposed method were certified with the MDA calibration curve. The results (Table 2) show that the recoveries range from 94.10% to 106.76%.

## 4. Conclusions

A novel molecularly imprinted electrochemical (MIEC) sensor with high selectivity and sensitivity was proposed for MDA detection. The results showed that the electrode modification with MWCNTs significantly enhanced the surface area and the conductivity of the electrode, resulting in improved sensitivity. The use of nanoparticles was also beneficial because of their antifouling properties. The electrodeposited MIP layer exhibited excellent electrochemical response thanks to CS’s exceptional film-forming ability and the bonding of CS to the target molecule. The sensor’s response to the MDA was linear, in the concentration range of 0.5 µM–100 μM, and the calculated LOD was 15 nM. This sensor displayed a low limit of detection, a wide linear concentration range, excellent selectivity, and satisfactory reproducibility. High recoveries (94.10 to 106.76%) were obtained with this sensor while detecting MDA in real samples. As a result, MIP/MWCNTs/GCE is a highly promising device for the monitoring trace amounts of MDA.

## Figures and Tables

**Table 1:** Comparison of the electrocatalytic oxidation peak current (*I*_p_) of MDA (500 µM) on various electrode surfaces at pH 10.0.

Electrode	Oxidation Peak Current (µA)	Drying Method
Bare GCE	1.13 ± 0.11	-
MIP/GCE	0.01 ± 0.003	IR lamp
NIP/MWCNTs/GCE	8.05 ± 0.26	IR lamp
MIP/MWCNTs/GCE	29.47 ± 0.46	IR lamp

**Table 2:** Determination of MDA in the real sample using MIP/MWCNT-modified electrodes. Three replicates were made for each run.

Samples	Spiked (µM)	Found (µM)	RSD (%)	Recovery (%)
Laminate structure (PET/EVOH/PE) including a PU adhesive	0	-	-	-
	15	15.37	2.1	102.46
	30	32.03	1.5	106.76
	50	47.05	2.8	94.10

## Data Availability

Data will be made available on request.

## References

[1] Lambertini F., Di Lallo V., Catellani D., Mattarozzi M., Careri M., Suman M. (2014). Reliable liquid chromatography-mass spectrometry method for investigation of primary aromatic amines migration from food packaging and during industrial curing of multilayer plastic laminates. J. Mass Spectrom..

[2] Pezo D., Fedel M., Bosetti O., Nerín C. (2012). Aromatic amines from polyurethane adhesives in food packaging: The challenge of identification and pattern recognition using Quadrupole-Time of Flight-Mass Spectrometry. Anal. Chim. Acta.

[3] Kolado W., Balcerzak M. (2009). The examination of migration of primary aromatic amines from laminated plastic food packaging materials into food simulants by spectrophotometric method. Acta Aliment..

[4] Campanella G., Ghaani M., Quetti G., Farris S. (2015). On the origin of primary aromatic amines in food packaging materials. Trends Food Sci. Technol..

[5] Sanchis Y., Yusà V., Coscollà C. (2017). Analytical strategies for organic food packaging contaminants. J. Chromatogr. A.

[6] Ghaani M., Nasirizadeh N., Yasini Ardakani S.A., Zare Mehrjardi F., Scampicchio M., Farris S. (2016). Development of an electrochemical nanosensor for the determination of gallic acid in food. Anal. Methods.

[7] Papaioannou G.C., Karastogianni S., Girousi S. (2022). Development of an electrochemical sensor using a modified carbon paste electrode with silver nanoparticles capped with saffron for monitoring mephedrone. Sensors.

[8] Amor-Gutiérrez O., Costa-Rama E., Fernández-Abedul M.T. (2022). Paper-based enzymatic electrochemical sensors for glucose determination. Sensors.

[9] Tomassetti M., Pezzilli R., Prestopino G., Di Natale C., Medaglia P.G. (2022). Novel electrochemical sensors based on L-Proline assisted LDH for H_2_O_2_ determination in healthy and diabetic urine. Sensors.

[10] Hong J., Wang Y., Zhu L., Jiang L. (2020). An electrochemical sensor based on gold-nanocluster-modified graphene screen-printed electrodes for the detection of β-Lactoglobulin in milk. Sensors.

[11] Zhang X., Ju H., Wang J. (2008). Electrochemical Sensors, Biosensors and Their Biomedical Applications.

[12] Hanssen B.L., Siraj S., Wong D.K.Y. (2016). Recent strategies to minimise fouling in electrochemical detection systems. Rev. Anal. Chem..

[13] Rahman S., Bozal-Palabiyik B., Nur Unal D., Erkmen C., Siddiq M., Shah A., Uslu B. (2022). Molecularly imprinted polymers (MIPs) combined with nanomaterials as electrochemical sensing applications for environmental pollutants. Trends Environ. Anal. Chem..

[14] Hamed E.M., Li S.F.Y. (2022). Molecularly imprinted polymers-based sensors for Bisphenol-A: Recent developments and applications in environmental, food and biomedical analysis. Trends Environ. Anal. Chem..

[15] Wei J., Liu C., Wu T., Zeng W., Hu B., Zhou S., Wu L. (2022). A review of current status of ratiometric molecularly imprinted electrochemical sensors: From design to applications. Anal. Chim. Acta.

[16] Gui R., Jin H., Guo H., Wang Z. (2018). Recent advances and future prospects in molecularly imprinted polymers-based electrochemical biosensors. Biosens. Bioelectron..

[17] Malik A.A., Nantasenamat C., Piacham T. (2017). Molecularly imprinted polymer for human viral pathogen detection. Mater. Sci. Eng. C.

[18] Seguro I., Rebelo P., Pacheco J.G., Delerue-Matos C. (2022). Electropolymerized, Molecularly Imprinted Polymer on a Screen-Printed Electrode—A Simple, Fast, and Disposable Voltammetric Sensor for Trazodone. Sensors.

[19] Wang B., Hong J., Liu C., Zhu L., Jiang L. (2021). An electrochemical molecularly imprinted polymer sensor for rapid β-Lactoglobulin detection. Sensors.

[20] Kadhem A.J., Gentile G.J., Fidalgo de Cortalezzi M.M. (2021). Molecularly imprinted polymers (MIPs) in sensors for environmental and biomedical applications: A review. Molecules.

[21] Zarejousheghani M., Rahimi P., Borsdorf H., Zimmermann S., Joseph Y. (2021). Molecularly imprinted polymer-based sensors for priority pollutants. Sensors.

[22] Maduraiveeran G. (2022). Nanomaterials-based portable electrochemical sensing and biosensing systems for clinical and biomedical applications. J. Anal. Sci. Technol..

[23] Suh J.-K.F., Matthew H.W.T. (2000). Application of chitosan-based polysaccharide biomaterials in cartilage tissue engineering: A review. Biomaterials.

[24] Lian W., Liu S., Yu J., Li J., Cui M., Xu W., Huang J. (2013). Electrochemical sensor using neomycin-imprinted film as recognition element based on chitosan-silver nanoparticles/graphene-multiwalled carbon nanotubes composites modified electrode. Biosens. Bioelectron..

[25] Yang Y., Fang G., Liu G., Pan M., Wang X., Kong L., He X., Wang S. (2013). Electrochemical sensor based on molecularly imprinted polymer film via sol–gel technology and multi-walled carbon nanotubes-chitosan functional layer for sensitive determination of quinoxaline-2-carboxylic acid. Biosens. Bioelectron..

[26] Srivastava J., Singh M. (2016). A biopolymeric nano-receptor for sensitive and selective recognition of albendazole. Anal. Methods.

[27] Li H.X., Yao W., Wu Q., Xia W.S. (2014). Glucose molecularly imprinted electrochemical sensor based on chitosan and nickel oxide electrode. Adv. Mater. Res..

[28] European Commission (2011). Commission Regulation (EU), No. 10/2011 of 14 January, 2011. Off. J. Eur. Union.

[29] Rao H., Chen M., Ge H., Lu Z., Liu X., Zou P., Wang X., He H., Zeng X., Wang Y. (2017). A novel electrochemical sensor based on Au@PANI composites film modified glassy carbon electrode binding molecular imprinting technique for the determination of melamine. Biosens. Bioelectron..

[30] Rezaei B., Hamedian Esfahani M., Ensafi A.A. (2016). Modified Au Nanoparticles/Imprinted Sol-Gel/Multiwall Carbon Nanotubes Pencil Graphite Electrode as a Selective Electrochemical Sensor for Papaverine Determination. IEEE Sens. J..

[31] Yun Y., Pan M., Fang G., Yang Y., Guo T., Deng J., Liu B., Wang S. (2017). Molecularly imprinted electrodeposition o-aminothiophenol sensor for selective and sensitive determination of amantadine in animal-derived foods. Sens. Actuators B Chem..

[32] Kor K., Zarei K. (2016). Development and characterization of an electrochemical sensor for furosemide detection based on electropolymerized molecularly imprinted polymer. Talanta.

[33] Zhao W.-R., Kang T.-F., Lu L.-P., Shen F.-X., Cheng S.-Y. (2017). A novel electrochemical sensor based on gold nanoparticles and molecularly imprinted polymer with binary functional monomers for sensitive detection of bisphenol A. J. Electroanal. Chem..

[34] Chen N., Chen L., Cheng Y., Zhao K., Wu X., Xian Y. (2016). Molecularly imprinted polymer grafted graphene for simultaneous electrochemical sensing of 4, 4-methylene diphenylamine and aniline by differential pulse voltammetry. Talanta.

[35] Skoog D.A., West D.M., Crouch S.R., Holler F.J. (2014). Fundamentals of Analytical Chemistry.

[36] Chen L., Lian H.-T., Sun X.-Y., Liu B. (2017). Sensitive detection of L-5-hydroxytryptophan based on molecularly imprinted polymers with graphene amplification. Anal. Biochem..

